# Supplementary material for: Isolation and transcriptomic analysis of Anopheles gambiae oenocytes enables the delineation of hydrocarbon biosynthesis
Source: eLife. 2020 Jun 15;9:e58019. doi: 10.7554/eLife.58019 (PMC7351493; doi:10.7554/eLife.58019)
Supplement: Supplementary file 2. — Their differential expression (Log2Fold change) compared to female carcass cells is also shown. Genes above the double line are within the 200 most highly expressed genes. [file elife-58019-supp2.docx]

| Gene | RNAseq Normalized counts in Female Oenocytes | Log_2_Fold Change  (all at p-value<0.001) |
| --- | --- | --- |
| Cyp4G16 | 266337 | 3,29 |
| Fatty acid synthase AGAP001899 | 105057 | 3,44 |
| Cyp4G17 | 93677 | 3,26 |
| Elongase AGAP007264 | 34216 | 3,16 |
| Fatty acid synthase AGAP028049 | 22062 | 3,37 |
| Fatty acid Reductase AGAP004787 | 21437 | 2,87 |
| Elongase AGAP013094 | 17688 | 3,73 |
| Propionyl-CoA synthetase AGAP001473 | 15219 | 3,31 |
| Desaturase AGAP003050 | 15068 | 3,03 |
| Fatty acid Reductase AGAP005984 | 13904 | 2,60 |
| Fatty acid Synthase AGAP008468 | 13811 | 3,33 |
| Fatty acid Reductase AGAP005986 | 13041 | 3,02 |
| Elongase AGAP003197 | 11810 | 3,52 |
| Elongase AGAP003195 | 9596 | 3,49 |
| Elongase AGAP003196 | 7688 | 3,36 |
| Fatty acid Reductase AGAP004784 | 7504 | 3,41 |
| Fatty acid Reductase AGAP005985 | 7160 | 1,85 |
| Elongase AGAP001097 | 6026 | 3,36 |
| Elongase AGAP005512 | 5523 | 3,05 |
| Elongase AGAP013219 | 5099 | 3,84 |
| Elongase AGAP004372 | 4785 | 2,92 |
