# Supplementary material for: Isolation and transcriptomic analysis of Anopheles gambiae oenocytes enables the delineation of hydrocarbon biosynthesis
Source: eLife. 2020 Jun 15;9:e58019. doi: 10.7554/eLife.58019 (PMC7351493; doi:10.7554/eLife.58019)
Supplement: Supplementary file 3. — Their differential expression (Log2Fold change) compared to male carcass cells is also shown. Genes above the double line are within the 200 most highly expressed genes. [file elife-58019-supp3.docx]

| Gene | RNAseq Normalized counts in Male Oenocytes | Log_2_Fold Change  (all at p-value<0.001) |
| --- | --- | --- |
| Cyp4G16 | 364580 | 2,83 |
| Cyp4G17 | 137776 | 3,23 |
| Fatty acid synthase AGAP001899 | 97839 | 3,08 |
| Elongase AGAP007264 | 49263 | 3,08 |
| Fatty acid synthase AGAP028049 | 25170 | 2,95 |
| Propionyl-CoA synthetase AGAP001473 | 23730 | 3,22 |
| Fatty acid Reductase AGAP004787 | 22177 | 3,02 |
| Fatty acid Synthase AGAP008468 | 21699 | 3,08 |
| Elongase AGAP013094 | 21692 | 3,00 |
| Desaturase AGAP003050 | 17972 | 2,99 |
| Fatty acid Reductase AGAP005986 | 14351 | 3,35 |
| Fatty acid Reductase AGAP005984 | 14128 | 2,48 |
| Elongase AGAP003197 | 10167 | 2,89 |
| Elongase AGAP003195 | 7320 | 2,74 |
| Elongase AGAP001097 | 6301 | 2,78 |
| Fatty acid Reductase AGAP004784 | 5009 | 2,73 |
| Fatty acid Reductase AGAP005985 | 3459 | 3,07 |
| Elongase AGAP004372 | 2932 | 1,91 |
| Elongase AGAP005512 | 2762 | 2,42 |
| Elongase AGAP003196 | 2248 | 2,34 |
| Elongase AGAP013219 | 753 | 2,45 |
