# Supplementary material for: Isolation and transcriptomic analysis of Anopheles gambiae oenocytes enables the delineation of hydrocarbon biosynthesis
Source: eLife. 2020 Jun 15;9:e58019. doi: 10.7554/eLife.58019 (PMC7351493; doi:10.7554/eLife.58019)
Supplement: Supplementary file 5. — Number of raw reads produced for each sample and number of reads after quality control. [file elife-58019-supp5.docx]

| **Sample** | **Sex** | **Tissue** | **# raw reads** | **# reads postQC** | **% reads postQC** |
| --- | --- | --- | --- | --- | --- |
| Sample_1-1F | Female | Oenocytes | 33819432 | 32551857 | 96.25% |
| Sample_2-2F | Female | Oenocytes | 33113392 | 31896623 | 96.33% |
| Sample_3-3F | Female | Oenocytes | 39251164 | 38036798 | 96.91% |
| Sample_4-4M | Male | Oenocytes | 32539976 | 31437568 | 96.61% |
| Sample_5-5M | Male | Oenocytes | 25424014 | 24436348 | 96.12% |
| Sample_6-6M | Male | Oenocytes | 40072886 | 39086446 | 97.54% |
| Sample_7-7BCF | Female | Carcass | 33445880 | 32562341 | 97.36% |
| Sample_8-8BCF | Female | Carcass | 53419358 | 52054378 | 97.44% |
| Sample_9-9BCF | Female | Carcass | 38663816 | 37832559 | 97.85% |
| Sample_10-10BCM | Male | Carcass | 41441984 | 40404352 | 97.50% |
| Sample_11-11BCM | Male | Carcass | 38099604 | 37047825 | 97.24% |
| Sample_12-12BCM | Male | Carcass | 42194602 | 41132109 | 97.48% |
| TOTAL | - | - | 451486108 | 438479204 | 97.12% |
